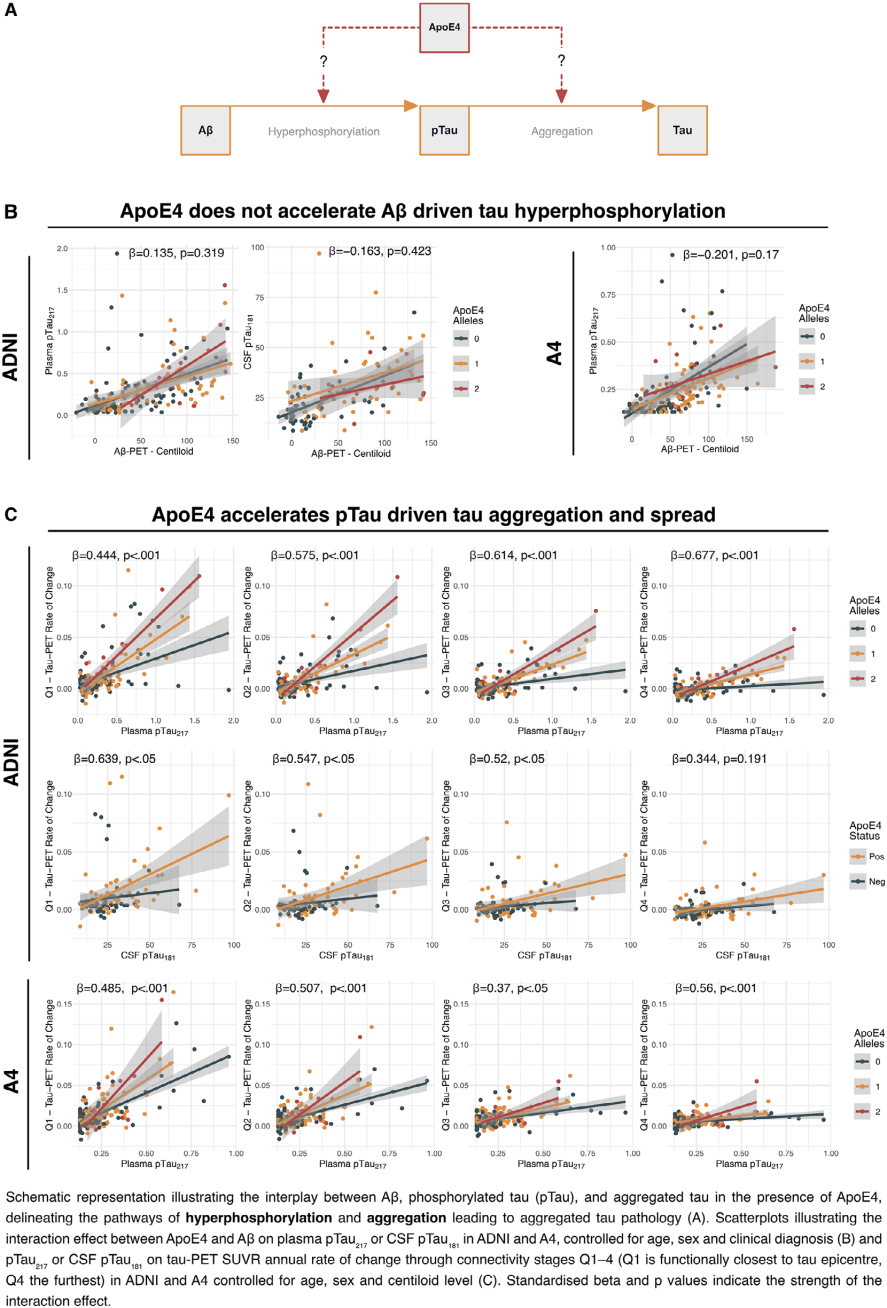# ApoE4 accelerates *p*‐tau driven tau aggregation and spread in Alzheimer's Disease in a allele‐dose dependent manner

**DOI:** 10.1002/alz70856_105476

**Published:** 2026-01-08

**Authors:** Anna Steward, Anna Dewenter, Sebastian Roemer‐Cassiano, Davina Biel, Zeyu Zhu, Lukas Frontzkowski, Fabian Hirsch, Madleen Klonowksi, Johannes Gnörich, Amir Dehsarvi, Matthias Brendel, Nicolai Franzmeier

**Affiliations:** ^1^ Institute for Stroke and Dementia Research (ISD), University Hospital, LMU Munich, Munich, Bavaria, Germany; ^2^ Max Planck School of Cognition, Leipzig, Sachsen, Germany; ^3^ Institute for Stroke and Dementia Research (ISD), University Hospital, LMU Munich, Munich, Germany; ^4^ Department of Nuclear Medicine, University Hospital, LMU Munich, Munich, Germany; ^5^ Department of Nuclear Medicine, University Hospital, LMU Munich, Munich, Bavaria, Germany; ^6^ Munich Cluster for Systems Neurology (SyNergy), Munich, Bavaria, Germany; ^7^ German Center for Neurodegenerative Diseases (DZNE), Munich, Germany; ^8^ Institute for Stroke and Dementia Research (ISD), LMU University Hospital, LMU, Munich, Bavaria, Germany; ^9^ Department of Psychiatry and Neurochemistry, University of Gothenburg, Mölndal, Västra Götalands län, Sweden

## Abstract

**Background:**

Understanding factors influencing Alzheimer's disease (AD) progression is crucial for optimising treatment timing and targets. A major genetic risk factor, the Apolipoprotein E ε4 allele (ApoE4), is associated with earlier tau pathology accumulation and spread at lower amyloid‐beta (Aβ) levels (Steward, JAMA Neurol, 2023). However, the mechanisms underlying this association remain unclear (Figure 1A). Therefore, we assessed how ApoE4 accelerates Aβ‐related tau aggregation. Specifically, we investigated whether ApoE4 promotes Aβ‐driven secretion of phospho tau (*p*‐tau) or ptau dependent tau aggregation, and determined whether ApoE4 promotes tau pathology in an allele dose‐dependent manner.

**Method:**

We analysed data from *APOE*‐genotyped AD‐spectrum participants in the ADNI (*n* = 201) and A4 cohorts (*n* = 200), integrating cross‐sectional fluid biomarker measures (plasma ptau_217_, CSF ptau_181_) and longitudinal Flortaucipir tau‐PET and Florbetaben/Florbetapir amyloid‐PET. Using linear regression, we assessed whether the interaction between amyloid‐PET and ApoE4 allele dosage influences plasma ptau_217_, and replicated this analysis with CSF ptau_181_ in an ADNI subset (*n* = 115). Secondly, to investigate whether ApoE4 enhances tau fibrilisation and spread, we calculated annual tau‐PET SUVR accumulation rates across a connectivity‐based tau spreading stages, using our prior methodology (e.g. Franzmeier, Sci Adv, 2020). Linear regressions tested the interaction between ptau_217_ (or CSF ptau_181_) and ApoE4 allele count on connectivity‐mediated tau‐PET accumulation in four connectivity stages that capture progressive tau spread.

**Result:**

ApoE4 allele dosage did not moderate the relationship between amyloid‐PET and plasma ptau_217_ in either sample (Figure 1B, ADNI: β=0.13, *p* = 0.32; A4: β=‐0.20, *p* = 0.17) nor between amyloid‐PET and CSF ptau_181_ in ADNI subsample (Figure 1B, b=‐.16, *p* = 0.42). However, a significant ApoE4 allele dose effect was observed in moderating the relationship between plasma ptau_217_ and tau‐PET accumulation across connectivity stages independent of amyloid burden (Figure 1C, ADNI: Q1–4 mean β=0.44, Q1‐4 *p* <0.001; A4: Q1‐4 mean β = 0.56, Q1,2,4 *p* <0.001, Q3 *p* <0.05), with the strongest effect in individuals carrying two ApoE4 alleles.

**Conclusion:**

ApoE4 exerts an allele dose‐dependent effect on ptau induced tau aggregation, driving accelerated tau spreading at lower Aβ levels. This suggests that attenuating soluble ptau increases in ApoE4 carriers may mitigate downstream tau fibrilisation and delay dementia onset, highlighting the potential of personalised therapeutic approaches.